# Acquired Perforating Dermatosis With Giant Lesions on the Scalp: A Unique Presentation in a Hemodialysis Patient

**DOI:** 10.7759/cureus.82850

**Published:** 2025-04-23

**Authors:** Raneem Alshahrani, Nasser Almulhim, Muneerah Alzouman, Shaikah Al-Aojan

**Affiliations:** 1 College of Medicine, Princess Nourah Bint Abdulrahman University, Riyadh, SAU; 2 College of Medicine, King Faisal University, Al Ahsa, SAU; 3 Department of Histopathology, Prince Sultan Military Medical City, Riyadh, SAU; 4 Department of Dermatology, Prince Sultan Military Medical City, Riyadh, SAU

**Keywords:** acquired perforating dermatosis, chronic renal failure, diabetes mellitus, hemodialysis, hyperkeratotic papules

## Abstract

Acquired perforating dermatosis is a rare skin disease characterized by umbilicated hyperkeratotic papules or nodules and transepidermal elimination of dermal components such as collagen and elastic fibers. It has been reported in association with various systemic disorders, particularly chronic renal failure and diabetes mellitus. Common locations include the extensor extremities and trunk. We describe a patient who developed multiple lesions on the scalp only, an unusual and rarely described location.

## Introduction

Acquired perforating dermatosis (APD) is the term used to describe perforating dermatosis affecting adult patients with systemic disease, most commonly diabetes mellitus and chronic kidney disease, often in the setting of dialysis [1]. It is characterized histologically by the transepidermal elimination of degenerated dermal material such as collagen, elastin, or fibrin and clinically by umbilicated hyperkeratotic papules or nodules over the extensor extremities [2]. We present a case of giant APD affecting the scalp solely in a patient with diabetes mellitus and chronic kidney disease on hemodialysis. 

This case was previously presented as a poster at the European Academy of Dermatology and Venereology Conference, held in Malta from 16-18 May 2024. 

## Case presentation

**Figure 1:**
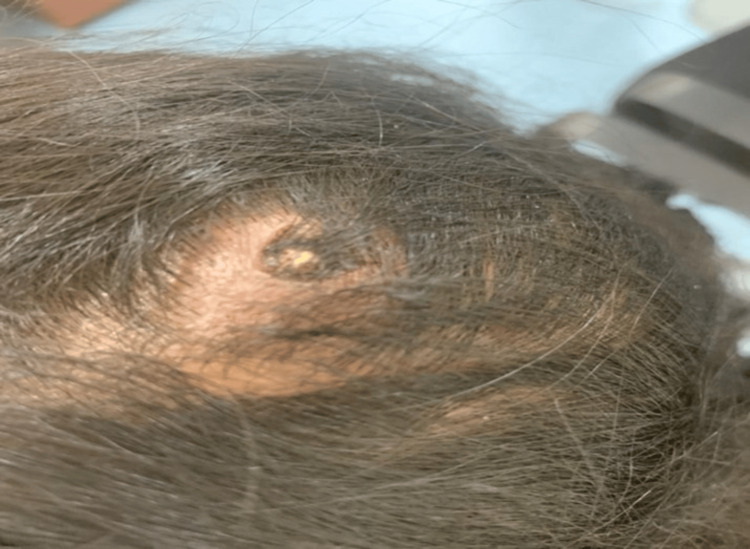
Firm, crusted, erythematous nodule with keratotic plug over the scalp.

**Figure 2:**
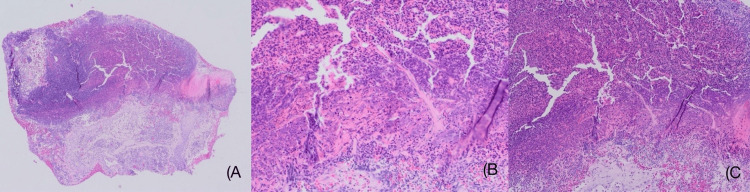
Epidermal ulceration with exuberant neutrophilic infiltration and transepidermal extrusion of vertically aligned collagen fibers. (A) Hematoxylin and eosin, original magnification 10x. (B) and (C) Hematoxylin and eosin, original magnification 20x.

**Figure 3:**
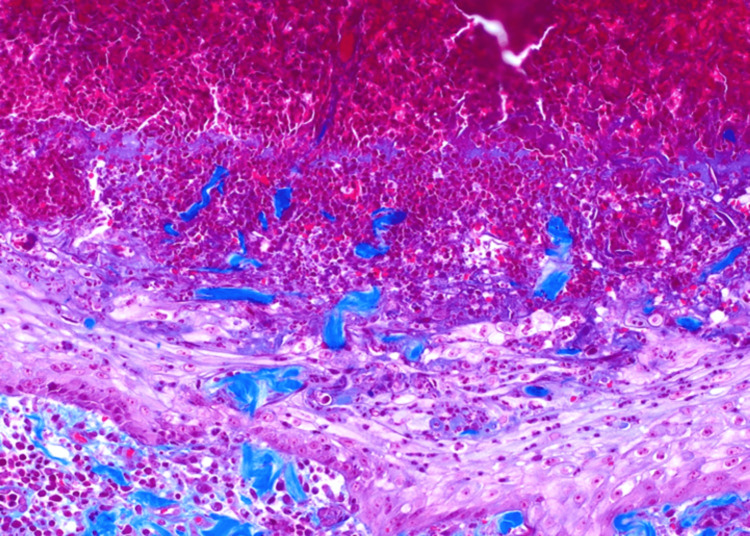
Transepidermal extrusion of vertically aligned degenerating collagen fiber, stained blue. (Masson trichrome stain, original magnification 200X).

**Figure 4:**
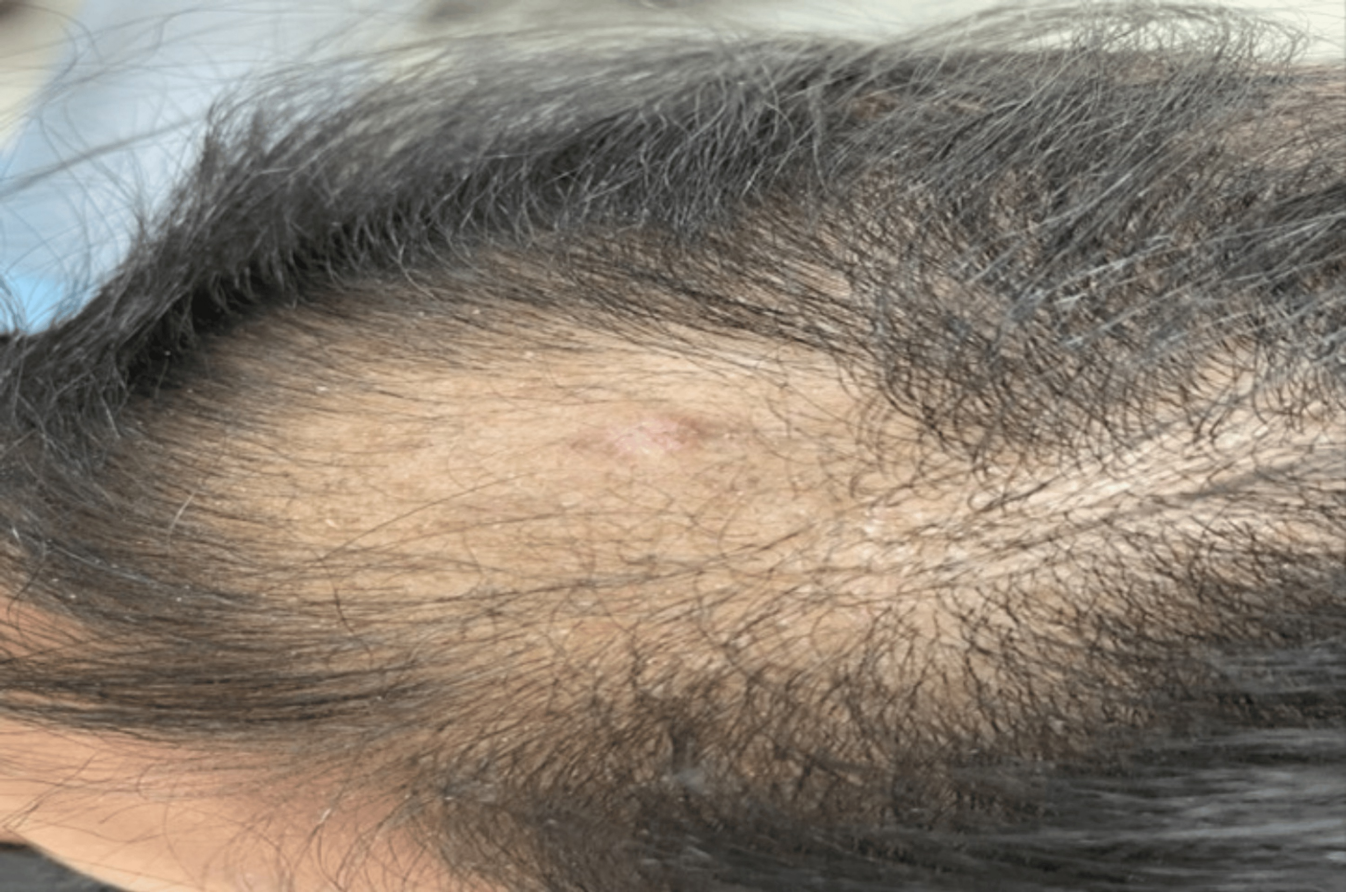
The lesion after treatment with intralesional triamcinolone acetonide 5 mg\cc injections.

A 30-year-old female presented to the dermatology clinic with itchy and mildly painful skin lesions over the scalp for two weeks. Skin lesions started abruptly with no history of preceding trauma. She had a previous similar episode two years ago. Medical history includes diabetes mellitus on insulin and end-stage renal disease on hemodialysis. Skin examination revealed five tender, firm, crusted papules and nodules with keratotic plugs scattered on the scalp (Figure 1). A skin punch biopsy was performed, which revealed epidermal ulceration with exuberant neutrophilic infiltration and transepidermal extrusion of vertically aligned collagen fibers (Figures 2a-2c). Masson's trichrome demonstrates the transepidermal extrusion of vertically aligned degenerating collagen fibers (Figure 3). The patient was diagnosed with APD and received intralesional triamcinolone acetonide 5 mg\cc injections, with dramatic improvement after the first session (Figure 4).

## Discussion

Acquired perforating dermatosis (APD) is a term coined by Rapini in 1989 to describe a group of acquired disorders characterized by transepidermal elimination of degenerated dermal material in patients with systemic disease [1]. Patients usually present with hyperkeratotic papules or nodules with a central crater filled with crust on the trunk and extensor surfaces, occasionally in linear distribution [3]. Lesions usually spare the face and mucous membranes [3]. González-Lara et al. reported a series of eight cases of APD, characterized by multiple umbilicated and pruritic papules with central keratotic plug [4]. The lesions predominantly affected the trunk and lower limbs [4]. However, presentation over the scalp only, as seen in our patient, is very unusual. The most severe perforating dermatoses have been observed in adult patients with chronic renal failure and diabetes mellitus [1]. Giant forms have also been reported in patients with diabetic nephropathy [2]. In a previous study, significant deterioration of renal function was observed in all patients diagnosed with APD during the attack of skin lesions [4]. On the other hand, our patient didn’t exhibit any changes in the renal profile during the onset of APD. Furthermore, APD has been described in association with hypothyroidism, hyperparathyroidism, liver disorders, neurodermatitis, and autoimmune diseases like systemic lupus erythematosus, vasculitis, dermatomyositis, lymphoma, breast carcinoma, or thyroid carcinoma [5]. The common factors among all of these pathologies are pruritus and scratching, and controlling pruritus helps clear the lesions [5]. The pathogenesis is not clear. However, the proposed mechanisms include altered differentiation of dermal components by glycosylation end products in diabetic patients and dialysis-related microdeposition of exogenous agents in chronic kidney disease patients [2]. Some of the treatments mentioned include topical and intralesional steroids, topical and systemic retinoids, cryotherapy, and ultraviolet radiation [3]. Allopurinol has been used in the management of giant forms [2]. Additionally, Özçelik et al. documented a marked improvement in the patient’s giant lesions after receiving 50 sessions of narrowband ultraviolet B (NB-UVB) therapy [6]. Similarly, our patient was treated with intralesional triamcinolone acetonide 5 mg/cc injections, leading to significant improvement after the first session.

APD is not usually considered in the differential diagnosis of scalp tenderness. Our case highlights the importance of considering this diagnosis, especially in patients with associated risk factors.

## Conclusions

The diagnosis of APD should be suspected in patients presenting with keratotic papules and nodules in unusual locations, such as the scalp. Especially if patients have associated risk factors such as diabetes mellitus and chronic kidney disease. Early recognition of its features in patients with strong associations will ensure timely diagnosis and management. Maintaining a broad differential diagnosis is essential to avoid misdiagnosis and to ensure optimal patient care.
